# The concept of the okadaic acid class of tumor promoters is revived in endogenous protein inhibitors of protein phosphatase 2A, SET and CIP2A, in human cancers

**DOI:** 10.1007/s00432-018-2765-7

**Published:** 2018-10-20

**Authors:** Hirota Fujiki, Eisaburo Sueoka, Tatsuro Watanabe, Masami Suganuma

**Affiliations:** 1 Faculty of Medicine, Saga University, Nabeshima, Saga 849-8501 Japan; 2 Graduate School of Science and Engineering, Saitama University, Saitama, 338-8570 Japan

**Keywords:** CIP2A, Okadaic acid, PP1, PP2A, SET, Tipα, TNF-α

## Abstract

**Purpose:**

The okadaic acid class of tumor promoters, which are inhibitors of protein phosphatases 1 and 2A (PP1 and PP2A), induced tumor promotion in mouse skin, rat glandular stomach, and rat liver. Endogenous protein inhibitors of PP2A, SET and CIP2A, were up-regulated in various human cancers, so it is vital to review the essential mechanisms of tumor promotion by the okadaic acid class compounds, together with cancer progression by SET and CIP2A in humans.

**Results and discussion:**

The first part of this review introduces the okadaic acid class compounds and the mechanism of tumor promotion: (1) inhibition of PP1 and PP2A activities of the okadaic acid class compounds; (2) some topics of tumor promotion; (3) *TNF-α* gene expression as a central mediator in tumor promotion; (4) exposure to the okadaic acid class of tumor promoters in relation to human cancer. The second part emphasizes the overexpression of SET and CIP2A in cancer progression, and the anticancer activity of SET antagonists as follows: (5) isolation and characterization of SET; (6) isolation and characterization of CIP2A; (7) progression of leukemia with SET; (8) progression of breast cancer with SET and CIP2A; (9) progression of lung cancer with SET; (10) anti-carcinogenic effects of SET antagonists OP449 and FTY720; and also (11) TNF-α-inducing protein of *Helicobacter pylori*, which is a clinical example of the okadaic acid pathway.

**Conclusions:**

The overexpression of endogenous protein inhibitors of PP2A, SET and CIP2A, is tightly linked to the progression of various human cancers, as well as Alzheimer’s disease.

## Introduction

**Fig. 1:**
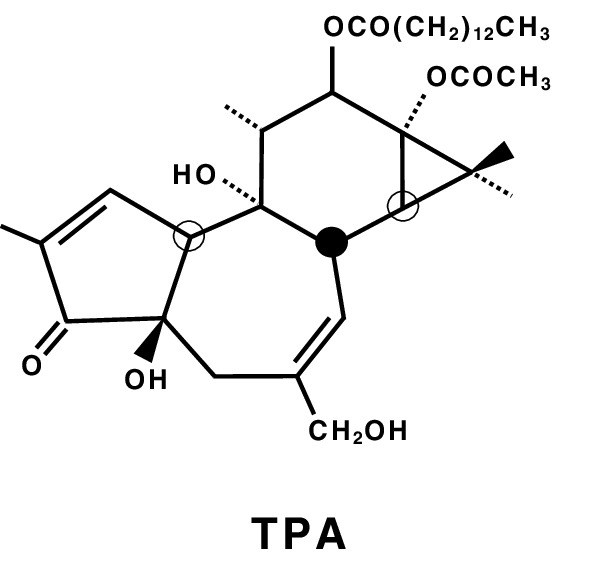
Structure of the classic tumor promoter, 12-*O*-tetradecanoylphorbol-13-acetate (TPA)

In 1915, Katsusaburo Yamagiwa and Koichi Ichikawa at the Imperial University of Tokyo reported pioneering evidence in their original experiments showing that continuous coal-tar painting on rabbit ears produced papillomas in 32 of 52 (62%) ears of the rabbits in over 70 days. They also paid special attention to the surface of ear skin: Before coal-tar painting, any dried coal-tar crust on the rabbits’ skin had to be frequently removed with tweezers from rabbit ears associated with inflammation and hyperemia. Yamagiwa called the skin “a carcinomatous medium” (Yamagiwa and Ichikawa 1915). This suggests that Yamagiwa had already conceived of tumor promotion and progression in the development of papillomas, based on the Reiztheorie (Irritation theory) of Rudolf Virchow (Fujiki 2014). The irritation theory has been revived in a standardized two-stage chemical carcinogenesis experiment with mouse skin by initiation with a single topical application of 7,12-dimethylbenz(a)anthracene (DMBA), and then repeated applications of a potent tumor promoter, 12-*O*-tetradecanoylphorbol-13-acetate (TPA), isolated from croton oil, *Croton tiglium* L. (Fig. 1) (Hecker et al. 1967). Although the significance of TPA in various science fields has engendered “the Renaissance of tumor promotion” (Hecker et al. 1984), topical applications of TPA or teleocidin, a TPA-type tumor promoter isolated from *Streptomyces mediocidicus* (Takashima and Sakai 1960), induced tumor promotion only in squamous cells of the skin, esophagus, and forestomach of mice in a transplacental initiation and postnatal promotion protocol (Goerttler et al. 1980; Suganuma et al. 1987).

**Fig. 2:**
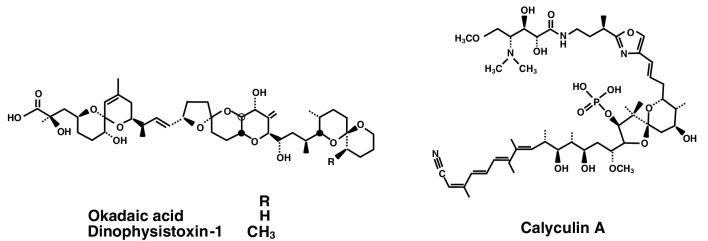
Structures of okadaic acid, dinophysistoxin-1 and calyculin A. Okadaic acid is a polyether derivative of a C_38_ fatty acid. Dinophysistoxin-1 is 35-methylokadaic acid. Calyculin A contains an octamethyl polyhydroxylated C_28_ fatty acid that is linked to two γ-amino acids and esterified by phosphoric acid

**Fig. 3:**
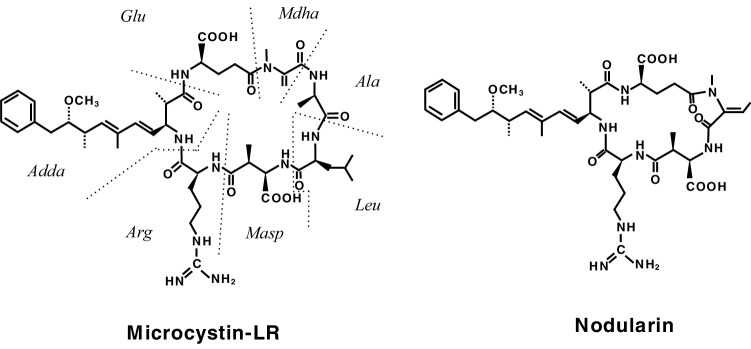
Structures of microcystin-LR and nodularin. Microcystin-LR contains, besides leucine and arginine, three d-amino acids and two unusual amino acids, 3-amino-9-methoxy-2,6,8-trimethyl-10-phenyldeca-4,6-dienoic acid (*Adda*) and *N*-methyl-dehydroalanine (*Mdha*). Nodularin is a monocyclic pentapeptide, which contains *Adda* but lacks one of the l- and d-amino acids found in the microcystins

Considering tumor promotion in human cancer, we aimed at finding potent new tumor promoters that do not activate protein kinase C (PKC) and have mechanisms different from the TPA-types. We were fortunate to receive okadaic acid from Japanese marine natural product chemists, along with their notes that okadaic acid might be a useful compound in cancer research. Thanks to their comments, we looked at a publication by Shoji Shibata showing that okadaic acid causes muscle contraction even in the absence of external Ca^2+^ ions (Shibata et al. 1982). The following three compounds are all tumor promoters as potent as TPA and teleocidin in mouse skin (Table 1): Okadaic acid, isolated from the black sponge *Halichondria okadai* (Fig. 2) (Tachibana et al. 1981), dinophysistoxin-1 (35-methylokadaic acid), isolated from the mussel *Mytidus edulis* (Fig. 2) (Murata et al. 1982), and calyculin A, isolated from a marine sponge *Discodermia calyx* (Fig. 2) (Kato et al. 1986). They all bound to okadaic acid receptors, the catalytic subunit of protein phosphatases 1 and 2A (PP1 and PP2A), and inhibited their activities (Table 2) (Suganuma et al. 1992a). It was exciting to find that okadaic acid in drinking water induced tumor-promoting activity in rat glandular stomach initiated with *N*-methyl-*N’*-nitro-*N*-nitrosoguanidine (MNNG) (Suganuma et al. 1992b). Moreover, microcystin-LR and nodularin, isolated from toxic blue–green algae *Cyanobacteria* (Fig. 3) (Carmichael et al. 1988; Rinehart et al. 1988) are potent inhibitors of PP1 and PP2A (Table 2) (Yoshizawa et al. 1990). Also, repeated i.p. injections of microcystin-LR or nodularin induced potent tumor-promoting activities in rat liver initiated with diethylnitrosamine (DEN) (Table 3) (Nishiwaki-Matsushima et al. 1992; Ohta et al. 1994). Okadaic acid, dinophysistoxin-1, calyculin A, microcystin-LR and nodularin all showed tumor-promoting activities in mouse skin, rat glandular stomach and rat liver, via the okadaic acid pathway by inhibition of PP1 and PP2A activities, so they are called the okadaic acid class of tumor promoters. The results strongly indicate that inhibitors of PP1 and PP2A activities may also induce tumor promotion and progression in various human cancers (Fujiki and Suganuma 1993).

**Table 1:** Tumor-promoting activities of the okadaic acid class and TPA-types, and activation of c-Ha-*ras* gene

Tumor promoters	Amounts per application (nmol)	Maximal % of tumor-bearing mice	Average No. of tumors per mouse in Week 30	Mutation of the second nucleotide in codon 61 of c-H-*ras*
Okadaic acid class				
Okadaic acid	1.2	86.7	7.2	A → T
Dinophysistoxin-1	1.2	100.0	8.5	A → T
Calyculin A	1.0	93.3	4.3	A → T
TPA-types				
TPA	4.1	100.0	11.0	A → T
Teleocidin	5.7	100.0	4.0	A → T

Fujiki and Suganuma (1993)

**Table 2:** Inhibition of protein phosphatase 1 and 2A activities by the okadaic acid class of tumor promoters and endogenous protein inhibitor of PP2A, SET

Inhibitors	Inhibition of PP1 IC_50_ (nM)	Inhibition of PP2A IC_50_ (nM)	References
Okadaic acid class			
Okadaic acid	3.4^a^	0.07^b^	Suganuma et al. (1992a)
Calyculin A	0.3^a^	0.13^b^	Suganuma et al. (1992a)
Microcystin-LR	0.1^a^	0.10^b^	Suganuma et al. (1992a)
Endogenous			
SET/I_2_^PP2A^		2.0^c^	Li et al. (1996)

^a^The catalytic subunit of PP1 was isolated from rabbit skeletal muscle (Brautigan and Shriner 1988)

^b^That  of PP2A was isolated from human erythrocytes (Brautigan and Shriner 1988)

^c^PP2A was isolated from bovine kidney cytosol (Amick et al. 1992)

**Table 3:**
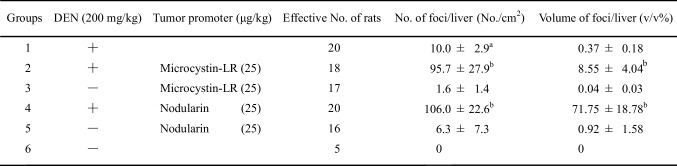
Induction of GST-P positive foci by microcystin-LR or nodularin

^a^Mean ± SD

^b^ *P* < 0.005 (Ohta et al. 1994)

Recently the genomic and cDNA cloning of a *dek-can* fusion gene in acute nonlymphocytic leukemia revealed a *set-can* fusion gene in translocation (6;9) (p23;q34), and the subsequent study of *set* gene showed that SET protein is an endogenous protein inhibitor of PP2A (von Lindern et al. 1992b; Li et al. 1995). The other endogenous inhibitor of PP2A - Cancer Inhibitor of PP2A (CIP2A) - was also isolated from human malignancies (Soo Hoo et al. 2002; Junttila et al. 2007). Since endogenous inhibitors of PP2A are physiological proteins found in various human cells, the overexpression of SET and CIP2A plays a vital part in causing cancer in humans. The mechanism of cancer progression with SET and CIP2A will probably be supported by studies of the okadaic acid pathway in rodent carcinogenesis, including hyperphosphorylation of proteins and gene expression of inflammatory cytokines. Moreover, the SET antagonists OP449 and FTY720 induced down-regulation of *set* gene expression, and thus reduced tumor growth (Janghorban et al. 2014; Agarwal et al. 2014). This review discusses these new topics: a brief introduction of okadaic acid class compounds; isolation and characterization of PP2A inhibitors, SET and CIP2A; progression of leukemia, breast and lung cancers with SET; anticancer effects of SET antagonists; and finally, TNF-α-inducing protein (Tipα) of *Helicobacter pylori* (*H. pylori*). This will help us to understand the molecular mechanism of SET and CIP2A overexpression in cancer cells. Tumor promotion among rodents with the okadaic acid class compounds is revived in human cancer progression by the endogenous protein inhibitors of PP2A, SET and CIP2A.

## Inhibition of PP 1 and PP2A activities of the okadaic acid class compounds

Okadaic acid inhibits protein phosphatase activity of purified PP1 and PP2A (Erdödi et al. 1988; Hescheler et al. 1988). Using our assay system for specific binding with [27-^3^H]okadaic acid to PP1 and PP2A, which are contained in both particulate and cytosolic fractions of mouse skin, we confirmed that dinophysistoxin-1, calyculin A, microcystin-LR and nodularin all bound to their catalytic subunits (Suganuma et al. 1989). We also found that the radioactive photoaffinity probe, [27-^3^H]methyl 7-*O*-(4-azidobenzoyl) okadaate, covalently bound to the catalytic subunit, but not to the two regulatory subunits of PP2A (Nishiwaki et al. 1990). Next, three representative classes of compound - okadaic acid, calyculin A and microcystin-LR—were determined to be inhibitors of the purified catalytic subunits of PP1 and PP2A under the same experimental conditions. Okadaic acid inhibits PP2A more potently than it inhibits PP1 (IC_50_s of 0.07 nM and 3.4 nM, respectively), and microcystin-LR is the strongest inhibitor of both PP1 and PP2A with the same potency (Table 2). The compounds did not show any significant inhibition of rat brain protein tyrosine phosphatase 1 (Ingebritsen 1991), so the okadaic acid class compounds are potent inhibitors of PP1 and PP2A, with slight differences (Suganuma et al. 1992a). The inhibitory activity of SET will be explained in another chapter.

## Some topics of tumor promotion

**Fig. 4:**
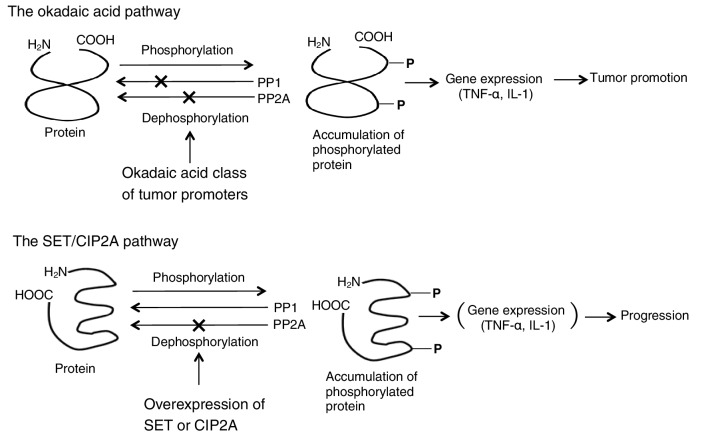
Schematic illustration of the okadaic acid pathway and the SET/CIP2A pathway

Based on evidence that okadaic acid and TPA both induced clonal expansion of initiated cells in mouse skin, though with different mechanisms (Table 1), we studied the effects of simultaneous applications of okadaic acid and teleocidin on mouse skin initiated with DMBA in two-stage carcinogenesis experiments. Three independent experiments with different doses of both tumor promoters - three combinations (1.0 + 2.5, 0.1 + 0.25 and 1.0 + 0.25 µg/application for okadaic acid + teleocidin), or each dose of okadaic acid alone, or teleocidin alone—revealed that simultaneous repeated applications of okadaic acid and teleocidin did not show any synergistic or additive effects on tumor-promoting activity in mouse skin. This absence of synergistic effects was also confirmed in two systems: Induction of ornithine decarboxylase in mouse skin and protein phosphorylation in human keratinocytes. The results are important to understand that okadaic acid and teleocidin or TPA have a common mechanism for signal transduction in the targeted cells, after the okadaic acid pathway and the PKC pathway have advanced in the cells (Fig. 4) (Suganuma et al. 1993).

Since TPA and teleocidin did not induce any tumor promotion in the stomach of mice, it was important to determine whether okadaic acid would show any tumor-promoting activity in the stomach, as a causative agent of diarrhetic shellfish poisoning. We conducted a two-stage carcinogenesis experiment in glandular stomach of male SD rats initiated with MNNG in the drinking water for 8 weeks, followed by giving a solution of okadaic acid in drinking water as a tumor promoter. The percentages of neoplastic change-bearing rats in the groups treated with MNNG plus okadaic acid, MNNG alone, or okadaic acid alone were 75.0 (*P* < 0.05), 46.4 and 0%, respectively (Suganuma et al. 1992b). These results were presented at the first International Gastric Cancer Congress in Kyoto March 29-April 1, 1995, organized by Mitsumasa Nishi, Haruo Sugano and Toshio Takahashi (Fujiki et al. 1995).

Microcystin-LR and nodularin are potent inhibitors of PP1 and PP2A (Table 2). The two-stage carcinogenesis experiments in rat liver were conducted using a DEN, and tumor promotion was conducted by repeated i.p. administrations of microcystin-LR or nodularin twice a week for 10 weeks. The glutathione *S*-transferase placental form (GST-P) positive foci in the liver sections were immunohistochemically stained with anti-GST-P-antibody (Sato et al. 1984), and the induction of GST-P positive foci by microcystin-LR or nodularin was assessed as a marker of tumor-promoting activity in the liver based on the number of foci/cm^2^ in the liver and volume of foci/liver (v/v%) (Table 3). Nodularin increased induction of GST-P foci more strongly than did microcystin-LR, and showed a tumor-promoting activity much stronger than microcystin-LR. The results show that nodularin is a carcinogenic inhibitor of PP1 and PP2A with both initiating and tumor-promoting activities, whereas microcystin-LR has potent tumor-promoting activity but not initiating activity (Ohta et al. 1994). Nodularin was found to markedly reduce the testoterone level in the course of hepatocarcinogenesis experiments with DEN plus nodularin in Fischer 344 male rats (Park et al. 2002).

## *TNF-α* gene expression as a central mediator in tumor promotion

*TNF-α* gene expression is strongly linked to tumor promotion by the okadaic acid class compounds in rodents. This has been supported by a report that okadaic acid mimicked the TNF-α- or IL-1-induced phosphorylation pattern of over 140 proteins (Guy et al. 1992). To demonstrate the importance of TNF-α in tumor promotion, we studied *TNF-α* gene expressions with both the liver tumor promoter nodularin and the mouse skin tumor promoter TPA - which is not a liver tumor promoter (Kitagawa et al. 1984). We found that nodularin induced dose-dependent expression of *TNF-α* gene in primary cultured rat hepatocytes, and also TNF-α release into the medium, whereas TPA did not induce any significant expression of *TNF-α* gene (Sueoka et al. 1997). The results strongly indicated that induction of *TNF-α* gene expression by a tumor promoter in vitro is similar to tumor-promoting activity in vivo (Fig. 4).

Discovery of the transforming activity with human TNF-α in BALB/3T3 cells encouraged us to move on to an experiment with the skin of TNF-α-deficient (TNF^−/−^) mice (Marino et al. 1997): Treatment with DMBA plus okadaic acid showed no tumors for up to 19 weeks in TNF^−/−^ mice, whereas the percentage of tumor-bearing TNF^+/+^ mice was 100%. Treatment with DMBA plus TPA delayed tumor onset in TNF^−/−^ mice 4 weeks, and the time to develop to 100% was 9 weeks later than that in TNF^+/+^ mice. We also found that residual tumor-promoting activity in skin of TNF^−/−^ mice was induced by *IL-1α* and *IL-1β* gene expression (Suganuma et al. 1999).

**Fig. 5:**
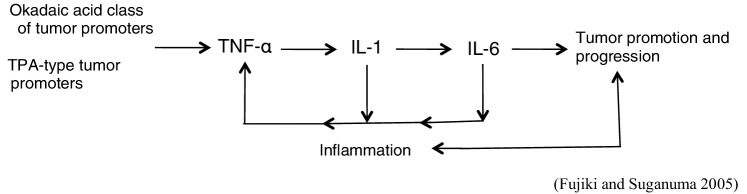
Sequence of cytokine cascade in tumor promotion

Further experiments with the skin of IL-6^−/−^ or IL-6^+/+^ C57BL/6 mice treated with DMBA plus okadaic acid, or DMBA plus TPA, revealed that the IL-6^−/−^ and IL-6^+/+^ groups did not show any significant difference in their tumor-promoting activities. Since IL-6 acts differently from TNF-α, and can be replaced by other cytokines, we think that TNF-α is the first endogenous instigator for the sequence of cytokine cascade from IL-1 to IL-6 and subsequently returns to TNF-α in tumor promotion (Fig. 5) (Suganuma et al. 2002; Fujiki and Suganuma 2005).

## Exposure to the okadaic acid class of tumor promoters in relation to human cancer

Okadaic acid and dinophysistoxin-1 are natural products in our environment. Since they are causative agents of diarrhetic shellfish poisoning, and tumor promoters for rat glandular stomach, we confirmed that intubation of okadaic acid (more than 10 µg/0.2 ml sesame oil) into the stomach of rats caused diarrhea with an accumulation of a large volume of fluid in the stomach, small intestine, and colon (Suganuma et al. 1988). And oral administration of 10 µg okadaic acid or 10 µg dinophysistoxin-1 caused significant enhancement of bromodeoxyuridine (BrdU)-labeling indices in mice (Yuasa et al. 1994). Since okadaic acid and dinophysistoxin-1 accumulate in the hepatopancreas of mussels and shellfish, consumption of these organisms causes diarrhetic shellfish poisoning, which has been reported in several countries, including Japan, Chile, Norway, The Netherlands, and Spain (Murata et al. 1982). These compounds are threats to public healths and also to the shellfish industry in Japan, although the amount of toxin needed to induce illness by oral intake is small for humans, equivalent to 32 µg of dinophysistoxin-1 (Yasumoto et al. 1985). Government regulations by the National Institute of Health Sciences Japan in 1981 set the maximum allowable levels of toxins in shellfish meat at 0.05 mouse unit/g meat, which corresponds to 0.16 µg okadaic acid/g meat (Fujiki and Suganuma 1993). Therefore, humans can avoid both acute and chronic toxicities of okadaic acid and dinophysistoxin-1.

Similarly, the intake of liver tumor promoters, such as microcystins and nodularin, are thought to correlate with development of human liver cancer, but epidemiological evidence on the effect of continuous exposure to humans has not been widely studied. Therefore, exposure to the okadaic acid class of tumor promoters apparently is not directly related to human cancer.

## Isolation and characterization of SET

The study of SET oncoprotein was initiated with the identification of translocation (6;9) (p23;q34) in acute myeloblastic leukemia (AML) associated with formation of a highly consistent *dek-can* fusion gene translocated to the 6p- chromosome (von Lindern et al. 1992a). The 3′ part of *can* in acute undifferentiated leukemia was found to fuse to a novel gene that is distinct from a previously isolated *dek* gene. This novel gene was named *set* gene, which is located 5′ of the *set*-*can* translocation breakpoint. The *set-can* fusion gene encodes a 5-kb chimeric transcript whose nucleotide sequence predicts a 155 kDa SET-CAN fusion protein. It is important to note that *set* gene was expressed in all adult mouse tissues analyzed, indicating that SET protein has a general physiological function in the organism. A putative SET protein contains 277 amino acids with 32 kDa, and shows homology with the yeast nucleosome assembly protein NAP-1, suggesting that SET may be a nuclear protein (von Lindern et al. 1992b). The 39 kDa SET from human erythroleukemia K-562 cells was phosphorylated mainly on the serine residue in vivo, but phosphothreonine and phosphotyrosine were not detected. Subcellular localization of SET in HeLa and human osteogenic sarcoma cells was observed mainly in nuclei (Adachi et al. 1994).

Li’s group first isolated two heat-stable protein inhibitors of PP2A from extract of bovine kidney, and they were designated as I_1_^PP2A^ and I_2_^PP2A^. The purified preparations of I_1_^PP2A^ and I_2_^PP2A^ have ~ 30 and ~ 20 kDa, respectively, as estimated by SDS–PAGE, and they inhibited PP2A activity with ^32^P-labeled myelin basic protein, ^32^P-labeled histone H1, ^32^P-labeled pyruvate dehydrogenase complex, ^32^P-labeled phosphorylase and protamine kinase as substrates. The preparations of I_1_^PP2A^ and I_2_^PP2A^ were less effective on PP1, PP2B and PP2C, and also pyruvate dehydrogenase phosphatase. Kinetic analysis showed that I_1_^PP2A^ and I_2_^PP2A^ were noncompetitive and displayed Ki of 30 and 25 nM, respectively. Moreover, I_1_^PP2A^ and I_2_^PP2A^ are considered to be products of distinct genes (Li et al. 1995). Based on evidence that an N-terminal amino acid sequence of I_2_^PP2A^ showed 70% homology with SET, I_2_^PP2A^ was found to be a truncated form of SET, a largely nuclear protein that fuses to nucleoporin Nup214 in acute nonlymphocytic myeloid leukemia. Half-maximal inhibition of the phosphatase occurred at about 2 nM purified recombinant SET, which was similar to purified preparation of I_2_^PP2A^ (Table 2). These results showed that SET is a potent and specific inhibitor of PP2A (Li et al. 1996). When the inhibitory potency of SET/I_2_^PP2A^ was compared with those of okadaic acid, calyculin A and microcystin-LR (Table 2), SET was about 30-fold weaker than the okadaic acid class compounds. Since SET is a physiological endogenous inhibitor of PP2A, the amount of SET must be increased by overexpression of SET to cause cancer, and also to produce similar potency of inhibition by okadaic acid. How the overexpression of SET and CIP2A is induced, has not yet been widely reported.

## Isolation and characterization of CIP2A

CIP2A was first found as a 90 kDa protein (p90) in human hepatocellular carcinoma, and the expression of endogenous p90 was also observed in cancer cell lines such as HeLa and normal keratinocytes; human *p90* gene is localized in chromosome region 3q13.13. Indirect immunofluorescence analysis revealed that p90 protein is localized to the cytoplasm in cultured cells and mouse fetal liver, but not in adult liver. However, p90 protein was overexpressed in 55% of human gastric cancer tissues, and anti-p90 auto-antibodies were detected in patients with hepatocellular carcinoma (13.1%), but not in patients with chronic hepatitis and acute hepatitis, HBsAg carriers, or normal controls. Anti-p90 auto-antibodies were also detected in 3.3 and 5.0% patients with gastric and esophagus cancer, respectively, but not those with colon cancer (Soo Hoo et al. 2002). Junttila et al. isolated a novel putative PP2A-interacting protein, KIAA1542, from human cancer cells: It was identical to p90 cytoplasmic protein discovered by Soo Hoo et al. and later designated as a Cancerous Inhibitor of PP2A (CIP2A). Inhibitory activity of PP2A was shown in an experiment that CIP2A inhibited c-Myc-associated PP2A activity and protected c-Myc at serine 62 from dephosphorylation in human cells. However, the exact molecular mechanism that results in PP2A inhibition by CIP2A has not yet been clearly elucidated. CIP2A protein levels were very low in human epidermal keratinocytes, nontumorigenic mouse embryo fibroblasts, and immortalized NIH3T3 mouse fibroblasts, but CIP2A was overexpressed in HeLa cells, HT-1080 fibrosarcoma cells, head and neck squamous cell carcinoma and colorectal cancer. The results showed that CIP2A is expressed at low levels in most nonmalignant tissues, but its expression increases in malignant cells in vivo, where the effects are similar to those of the okadaic acid class compounds in various organs of rodents. Dense foci formation on a monolayer of HeLa cells transfected with CIP2A.1-targeted siRNA abrogated cell foci formation 10 days after transfection, and HeLa cells transfected with CIP2A-targeted siRNA reduced overall tumor size in xenograft mouse model, resulting in significant inhibition of tumor weight (Junttila et al. 2007; Böckelman et al. 2012).

## Progression of leukemia with SET

SET mRNA levels in B-cell chronic lymphocytic leukemia (CLL) were 11.2 ± 2.7-fold higher than the mean of normal B cells, and immunoblots from the same samples showed that both the α-isoform and the β-isoform of SET protein, which result from alternative splicing, were higher in CLL than in normal B cells. SET mRNA in Raji cells was 10.5 ± 0.7-fold higher than in normal B cells, and that in Ramos cells was 8.2 ± 0.4-fold higher than in normal B cells. Knockdown of SET in Raji cells after transfection with SET-targeted shRNA lentivirus resulted in a reduction of SET levels by 50% (Christensen et al. 2011a). Thus, overexpression of SET protein leads to progression of human cancer.

## Progression of breast cancer with SET and CIP2A

SET and CIP2A mRNA levels were high in breast cancer cells compared with the immortalized but nontransformed human breast epithelial cell line MCF10A. SET was overexpressed in about 50–60%, and CIP2A in about 90%, of breast cancers, and CIP2A overexpression was associated with triple negative, basal, and claudin-low tumor subtypes. When breast cancer cell lines MDA-MB-231, MDA-MB-436 and MDA-MB-468 were transfected with SET-targeted siRNA, CIP2A-targeted siRNA or scrambled siRNA, and then injected into the mammary gland of nonobese diabetic (NOD)/SCID/γ-chain null (NSG) mice, both SET knockdown and CIP2A knockdown reduced tumorigenic potential of breast cancer cell lines, compared with scrambled siRNA transfected cells (Janghorban et al. 2014). Therefore, SET protein induces progression of human breast cancer and may be a potential therapeutic target for treatment of breast cancer. The stable suppression of SET expression by lentivirus-mediated RNA interference (RNAi) inhibited the growth, migration and invasion of breast cancer cell lines MDA-MB-231 and ZR-75-30. In addition, knockdown of SET increased the expression of PP2Ac and PP2A activities, and also reduced MMP-9 expression in breast cancer cells compared with control cells (Li et al. 2014).

The overexpression of CIP2A mRNA was found in human breast cancers: 159 previously characterized human mammary tumors and 5 normal breast samples (*P* = 0.027). It is important to note that mucinous mammary carcinomas with a good prognosis displayed low expression levels of CIP2A mRNA, similar to those for normal breast samples, indicating that CIP2A expression is linked to poor prognosis of human breast cancer. Moreover, when MDA-MB-231 cells transfected with either CIP2A-targeted siRNA or scrambled siRNA were injected into the mammary fat pad of athymic mice, CIP2A depletion led to a significant decrease in tumor volume and weight, as measured at the end of the experiment. The results showed that CIP2A promotes malignant growth of human breast cancer cells (Côme et al. 2009).

## Progression of lung cancer with SET

Lung cancer is one of the leading causes of cancer deaths world-wide, and non-small cell lung cancer (NSCLC) accounts for approximately 80% of lung cancer diagnoses. SET expression in clinical samples of 163 NSCLC and 42 adjacent normal tissues was determined by immunohistochemistry analysis: SET was overexpressed in 91.4% of tumor samples (149/163), whereas the adjacent normal tissues exhibited undetectable or low SET staining, showing that SET is a significant molecule in lung cancer development. Clinicopathological analysis showed that NSCLC patients with high SET expression had poorer overall survival than those with low SET expression (*P* < 0.01). Knockdown of SET with SET-targeted siRNA significantly decreased cell viability and the BrdU positive cells in A549 and H460 cells, compared with those of control group. Thus, SET oncoprotein is a potential prognostic marker, and a new therapeutic target for NSCLC patients (Liu et al. 2015).

## Anti-carcinogenic effects of SET antagonists OP449 and FTY720

Endogenous protein inhibitors of PP2A, SET and CIP2A have led to a new strategy for treatment of cancer, based on evidence that SET and CIP2A knockdowns with siRNA or RNAi and SET antagonists, such as OP449 and FTY720, significantly reduced tumor growth. SET antagonists are here briefly discussed.

OP449 (formerly COG449) is a specific, physiologically stable, cell-penetrating peptide that binds to SET, and antagonizes SET’s inhibition of PP2A. OP449 is selectively cytotoxic to leukemic cell lines and primary patient cells with tyrosine kinase inhibitor-resistant BCR-ABL1 kinase mutations. Any increase in PP2A activity by OP449 efficiently and specifically inhibits growth of chronic myelogenous leukemia (CML). Treatment of AML cell line, MOLM-14 cells with 2.5 µM OP449 or 1 nM AC220 alone, a small molecule of the receptor tyrosine kinase inhibitor, resulted in cell viability reduced by 58 and 75%, respectively, and the combination of OP449 and AC220 synergistically reduced cell growth by almost 96%: The combination of OP449 and AC220 has a novel therapeutic efficacy for treatment of CML and AML (Agarwal et al. 2014). OP449 is a dimer of a chimeric peptide that is composed of an apoE-mimetic domain that binds to SET, which is fused to antennapedia, a protein transduction domain. The interaction of OP449 with SET causes release of SET from PP2A and increases PP2A activity, which was demonstrated in both leukemia cells and some solid tumor cell lines, including MDA-MB-231 (Switzer et al. 2011; Janghorban et al. 2014).

FTY720 (Fingolimod, Gilenya) is an oral sphingosine analog used in relapsed multiple sclerosis patients because of its immunosuppressive activity, which depends on lymphocyte sequestration in the lymph nodes. FTY720 undergoes phosphorylation (FTY720-P) by sphingosine kinase, and so acts as an immunosuppressant, that binds/internalizes the sphingosine-1-phosphate receptor. FTY720 also selectively induces apoptosis of neoplastic cells, but not normal cells: Treatment with FTY720 has resulted in toxicity-free long-term survival of leukemic animals in vivo (Oaks et al. 2013). Moreover, treatment with FTY720 markedly increased PP2A activity in A549 and H460 cells, inhibited NSCLC cell proliferation in a dose-dependent manner (Liu et al. 2015), and also showed an additive effect with 5-fluorouracil, SN-38, and oxaliplatin, which are drugs used in standard chemotherapy for patients with colorectal cancer (Cristóbal et al. 2014). However, the U.S. Food and Drug Administration found that FTY720 may in some cases mildly affect cardiac performance (Schmouder et al. 2012), and suggested that careful clinical use is recommended. In summary, endogenous protein inhibitors of PP2A, SET and CIP2A are physiologically important proteins in the cells, and the overexpression of SET and CIP2A induces strong inhibition of PP2A activity, resulting in tumor promotion and progression of human cancers, as shown by the okadaic acid pathway in rodent carcinogenesis experiments (Fig. 4). To better understand the molecular mechanism of progression by overexpression of PP2A inhibitors in cancer cells, Tipα of *H. pylori* is presented as an example.

## TNF-α-inducing protein of *H. pylori* can aid in understanding the mechanism of progression by endogenous PP2A inhibitors

In our previous review article of Adv. Cancer Res. in 1993, we considered three possibilities on how the okadaic acid pathway is related to human cancer: (1) exposure to the okadaic acid class compounds, which had already been reported; (2) involvement of endogenous protein inhibitors of PP1 and PP2A in the cells - this possibility is the main topics of this review article. Here we discuss a third possibility: that the effects of okadaic acid class compounds can be mimicked by those of cytokines, such as TNF-α and IL-1 (Fujiki and Suganuma 1993). Therefore, (3) the Tipα of *H. pylori* can provide insight into the molecular mechanisms of SET and CIP2A overexpression in human cancer cells.

*H. pylori* is classified as the definitive carcinogen for gastric cancer in humans (IARC Working Group 1994), and a gene product of *H. pylori* possessing TNF-α-inducing activity is thought to act as a tumor promoter for human gastric cancer. Since *Helicobacter pylori* membrane protein 1 (HP-MP1) can induce *TNF-α* gene expression in Bhas 42 (v-H-*ras* transfected BALB/3T3) cells (Yoshida et al. 1999; Suganuma et al. 2001), a gene (*HP0596*) similar to *HP-MP1* gene was identified in the genome sequence of *H. pylori* strain 26695 and designated as the *TNF-α-inducing protein* (*Tipα*) gene (Suganuma et al. 2005). Tipα is a protein of 172 amino acids with 19 kDa, and the dimer of Tipα with 38 kDa is an active form. In clinical practice, *H. pylori* bacteria obtained from 17 gastric cancer patients secreted Tipα in culture media significantly higher than did *H. pylori* from 11 chronic gastritis patients (Suganuma et al. 2008). Furthermore, we found that a specific binding protein for Tipα was nucleolin with 88 kDa, and that the direct binding complex of Tipα to nucleolin was incorporated into the cells of human gastric cancer cell line MKN-1 (Watanabe et al. 2010a, b).

The Cancer Signaling Phospho-antibody Array revealed that treatment of MKN-1 cells with Tipα showed strong phosphorylation of 11 cancer-related proteins, including MEK, ERK and MSK1, among 89 cancer-related proteins. However, phosphorylation of only two proteins was reduced at the same time. This suggests that Tipα increased protein phosphorylation, probably in the same way as by SET or CIP2A. Treatment of MKN-1 cells with nucleolin-targeted siRNAs down-regulated the expression of nucleolin in the membrane fraction, and inhibited the migration, adhesion and morphological changes induced by Tipα (Watanabe et al. 2014). Since nucleolin acts as a receptor for various molecules (Srivastava and Pollard 1999), the role of cell-surface nucleolin has now been further recognized as a central mediator for carcinogenic, anti-carcinogenic, and disease-related ligands (Fujiki et al. 2014).

## Discussion

We will briefly discuss SET in relation to inflammation, since inflammation is associated with various human diseases: Alzheimer’s disease (AD), for example, develops when abnormally phosphorylated or mutated tau loses affinity for microtubles and forms neurofibrillary tangles inside the cells (Kellogg et al. 2018). The relative expression of I_1_^PP2A^ and I_2_^PP2A^ (SET) mRNAs after normalization with GAPDH mRNA was ~ 25 (*P* < 0.001) and 10% (*P* < 0.05) higher, respectively, in AD temporal and entorhinal cortices than in the corresponding areas of the control cases. The 10 to 25% increase in expression of PP2A inhibitors could have a considerable accumulative effect over time on the hyperphosphorylation of tau and consequent neurofibrillary degeneration. AD is a slow but steadily progressive neurodegenerative disorder with an average progression of 7–10 years (Tanimukai et al. 2005). The apoE and apoE-mimetic peptide bound to the SET oncoprotein, which is associated with an increase in PP2A activity. Treatment with this apoE-mimetic peptide suppressed microglial activation and secretion of inflammatory mediators (e.g., TNF-α and IL-6 proteins) in primary microglial cultures and microglial cell lines: ApoE is an antagonist of SET protein (Christensen et al. 2011b). CIP2A-mediated PP2A inhibition drives tau/APP hyperphosphorylation and increases APPβ-cleavage and Aβ production (plaques) between neurons (Shentu et al. 2018). Both the enhancing endogenous PP2A activity and the antagonizing SET and CIP2A reduce levels of phosphorylated kinases and inflammatory response with TNF-α, IL-1 and IL-6. In summary, the okadaic acid class of tumor promoters are transformed into SET and CIP2A, which induce tumor promotion, progression and inflammation in various human cancer cells, which are the results of some life-style related diseases.
